# Supplementary material for: Acaricidal activity of plant extracts against Amblyomma variegatum in Waghimra, Northeastern Ethiopia
Source: PLoS One. 2026 May 11;21(5):e0348007. doi: 10.1371/journal.pone.0348007 (PMC13160357; doi:10.1371/journal.pone.0348007)
Supplement: Attachment — Submitted filename: Review Report_PONE-D-25-64821.docx [file pone.0348007.s002.docx]

**Review Report**

**Comments and Suggestions for Authors**

The paper title “Acaricidal Activity of Plant Extracts Against Amblyomma variegatum Tick Species in Waghimra, Northeastern Ethiopia” by Teklu Yitbarek et al.,. The manuscript discusses a key issue in veterinary parasitology and tick control with plant-based options, especially targeting Amblyomma variegatum, a significant tick in sub-Saharan Africa. The research idea is sound and aligns with the global trend toward biological control and plant-based alternatives. However, the study in its current form suffers from methodological, statistical, and editorial weaknesses that require substantial revision before acceptance for publication.

I thank the authors for their efforts in preparing this manuscript, which addresses a relevant topic in parasite control using plant-based, environmentally friendly alternatives.

**Title**

- The title is direct and good but delete the word “Tick Species” is unnecessary because the species is already defined.

**Abstract**

- The LC50 or LC90 value not mentioned, which is essential in toxicity studies
- The phrase “These findings support the reason for the traditional use", Please delete it
- There is no mention of the tick exposure period (7 days) for understanding the reported mortality rates.

**Introduction**

- What's new compared to previous studies? Is this the first study in Waghimra?
- Are A. variegatum ticks considered a new record in Waghimra?

**Materials and methods**

- Why was the study described as "cross-sectional" when it was experimental, not observational? Please review.
- Why was the study long-term (May 2023–September 2024)? Was it seasonal?
- The plants used in the study were not classified.
- Further clarification is needed in the Crud extraction method and storage section.
- Storing the extract for 5 months is excessive. Why was this statement included?
- Was there a positive control in the juvenile stage experiment?
- Statistical analysis: No probit regression was used to calculate the LC50.

**Results & Dissection**.

- The highest mortality rate (70%) is considered insignificant (low).
- There is a discrepancy in the results, where in the ANOVA table, it states "Dose = Not Significant (0.083)," yet the text mentions a "significant dose-dependent killing effect." Please clarify or correct this.
- The results were compared with studies on *Rhipicephalus microplus* and *Tetranychus urticae*. This is a scientific error because different species have different sensitivities. The discussion should first focus on the same species and then, in parallel, cite other species.
- 98% inhibition (percent control (PC)) was recorded, but there is no calculation of the Reproductive Index or Egg Hatchability Assessment.

I add some comments and suggestions for improvement to the manuscript. Finally, the manuscript can be accepted after a Major revision.

Best Regards,

Mohamed Baz

Associate Prof. Mohamed M. Baz

Medical Entomology

Department of Entomology, Faculty of Science,

Benha University. Benha 13518, Egypt

Email: mohamed.albaz@fsc.bu.edu.eg

002-01063070572

16 Feb., 2026
